# An Ex Vivo Morphometric Study of the Feline Corneal Endothelium (100 Eyes)

**DOI:** 10.1111/vop.70093

**Published:** 2025-10-28

**Authors:** Yamit Soueid, Netta Cremer, Noya Aharon, Bar Fruchter, Lionel Sebbag

**Affiliations:** ^1^ Koret School of Veterinary Medicine The Hebrew University of Jerusalem Rehovot Israel

**Keywords:** age‐related changes, corneal edema, endothelial cell density, polymegathism, translational research, vital staining

## Abstract

**Background:**

The feline corneal endothelium plays a critical role in maintaining corneal clarity, yet little is known about its baseline morphology and age‐related changes. This study aimed to assess endothelial morphology ex vivo using a vital dye‐based imaging technique, offering a practical tool for assessing endothelial health in settings where in vivo imaging may be impractical or unavailable.

**Methods:**

Central corneal buttons (8‐mm) were collected post‐mortem from 50 cats (100 eyes) and processed within 6 h of death or euthanasia. The endothelium was stained with 0.25% trypan blue and 0.5% alizarin red (pH 4.2) and imaged using light microscopy at 20× magnification. Morphometric analysis included mean cell density (MCD), mean cell area (MCA), polymegathism (coefficient of variation of cell area), and pleomorphism (% hexagonality). Statistical analyses assessed differences by age (< 10 vs. ≥ 10 years) and sex, with correlations to age explored via Spearman testing.

**Results:**

Median (±SEM; range) values across all eyes were as follows: MCA 352 ± 10 μm^2^ (104–458), MCD 2766 ± 196 cells/mm^2^ (2250–10 183), polymegathism 14% ± 0.3% (11–20), and pleomorphism 85% ± 0.8% (60–94). Older cats had significantly larger MCA (367 vs. 330 μm^2^; *p* < 0.001), lower MCD (2625 vs. 2970 cells/mm^2^; *p* < 0.001), and higher pleomorphism (86% vs. 82%; *p* =f). Age strongly correlated with MCD (*r* = −0.653, *p* < 0.001) and MCA (*r* = 0.593, *p* < 0.001).

**Conclusions:**

This ex vivo approach enables detailed evaluation of feline corneal endothelial morphology, revealing clear age‐related trends. The technique is practical and cost‐effective for veterinary and translational research, with potential to support studies of endothelial health and dysfunction across species.

## 1 Introduction

The corneal endothelium is a monolayer of hexagonal cells lining the posterior surface of the cornea, essential for preserving corneal transparency and overall ocular health [1]. Acting as a semi‐permeable barrier, this layer regulates fluid homeostasis and nutrient transport by pumping ions and metabolites between the aqueous humor and corneal stroma [2]. Given the close anatomical and physiological similarities between human and feline corneal endothelium [3, 4], cats offer a spontaneous and clinically relevant model for human endothelial disease such as age‐related endothelial decompensation or post‐operative endothelial failure following cataract or glaucoma surgery [1]. In particular, the limited mitotic capacity and age‐related cell loss in cats closely mirror human endothelial aging [5], offering a relevant platform for studying therapeutic interventions [3]. In felines, as in other mammals, the corneal endothelium is particularly vulnerable to damage from elevated intraocular pressure, intraocular inflammation, trauma, and a range of ocular and systemic diseases. Unlike regenerative species such as rabbits, feline endothelial cells have limited proliferative capacity, and significant cell loss results in irreversible structural changes and corneal endothelial decompensation [6, 7].

Following endothelial damage, the remaining cells attempt to restore continuity by undergoing compensatory hypertrophy and migration [8]. This reparative response leads to decreased cell density and abnormal cellular morphology, including pleomorphism (irregular cell shape) and polymegathism (variability in cell size) [9, 10]. Advanced imaging modalities, such as specular and confocal microscopy, have been employed to study the feline corneal endothelium in vivo [5, 11, 12, 13, 14]. Although these technologies provide valuable insights into endothelial cell morphology and density, their availability is often limited in veterinary settings due to cost and technical requirements. As a result, there remains a paucity of data on the normal structure and aging‐related changes of the feline corneal endothelium, hindering both clinical assessment and research.

To address this gap, the present study employed a vital dye‐based ex vivo method—recently described in other species [15, 16]—to evaluate corneal endothelial morphology and density in feline cadaveric eyes. By combining trypan blue and alizarin red staining with light microscopy, this approach enables detailed morphometric analysis at a relatively low cost, offering a practical and accessible alternative for corneal endothelial research. Accordingly, the study objectives were to (i) establish baseline morphometric parameters for the feline corneal endothelium, (ii) assess age‐related morphological changes, and (iii) evaluate the feasibility of this technique in feline eyes. These findings may facilitate future investigations into corneal endothelial health and disease, with potential applications in both veterinary and human medicine [3].

## 2 Materials and Methods

### 2.1 Sample Collection

Corneal samples were obtained post‐mortem from client‐owned cats that were euthanized or died due to unrelated medical conditions (e.g., heart failure, kidney disease). All cases were sourced through the University Teaching Hospital of the Koret School of Veterinary Medicine, with written informed consent provided by the owners or their representatives for the use of cadaver tissues in research. As the study involved only post‐mortem tissue collection with no prospective interventions or experimental procedures, it did not require formal approval by the institutional animal ethics committee, in accordance with institutional guidelines. Prior to tissue collection, slit‐lamp biomicroscopy (SL‐17, Kowa Medical Care) was performed by a trained examiner (YS, LS) to exclude eyes with overt anterior segment abnormalities. Within 6 h post‐mortem, full‐thickness central corneal buttons—including the epithelium, stroma, and endothelium—were excised using an 8‐mm biopsy punch. The samples were gently rinsed with balanced salt solution (BSS; Alcon Laboratories) to remove residual debris and blood, placed on a glass slide with the endothelial side up, and processed as described below.

### 2.2 Vital Dye Staining

**FIGURE 1:**
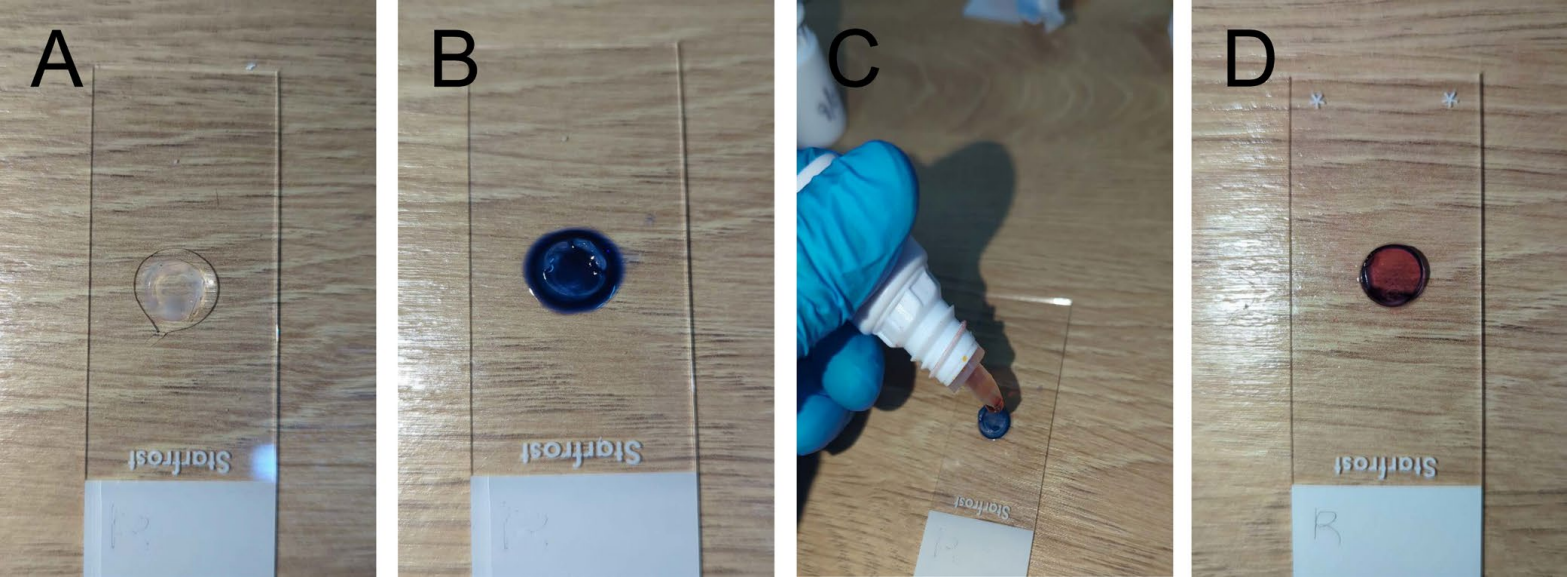
Stepwise staining procedure of a feline 8‐mm central corneal button using vital dyes. (A) Full‐thickness corneal button positioned on a glass slide with the endothelial surface facing up prior to staining. (B) Application of 0.25% trypan blue dye to the endothelial surface. (C) After 90 s, the cornea is gently rinsed off with balanced salt solution (BSS), followed by application of 0.5% alizarin red for an additional 90 s. (D) Final appearance of the cornea after a second rinse with BSS.

The endothelium was stained sequentially using two vital dyes as previously described in dogs and sheep [15, 16]. The first stain was trypan blue, prepared as a 0.25% solution by diluting 0.4% trypan blue (Sigma Aldrich) with sterile water for injection (Hospira Inc.). The second stain was alizarin red, prepared as a 0.5% solution with pH 4.2 by mixing the dye (Alizarin Red S, Sigma Aldrich) with sterile water for injection, then reaching the desired pH by adding minute amounts of hydrochloric acid (Detrex Chemicals). Both solutions were prepared fresh monthly, refrigerated at 4°C and handled by a pharmacist to ensure staining consistency. In each corneal sample, the endothelium was covered with 0.25% trypan blue solution for 90 s, gently rinsed with BSS, and then stained with 0.5% alizarin red for an additional 90 s before a final rinse with BSS (Figure 1).

### 2.3 Image Acquisition and Morphological Analysis

**FIGURE 2:**
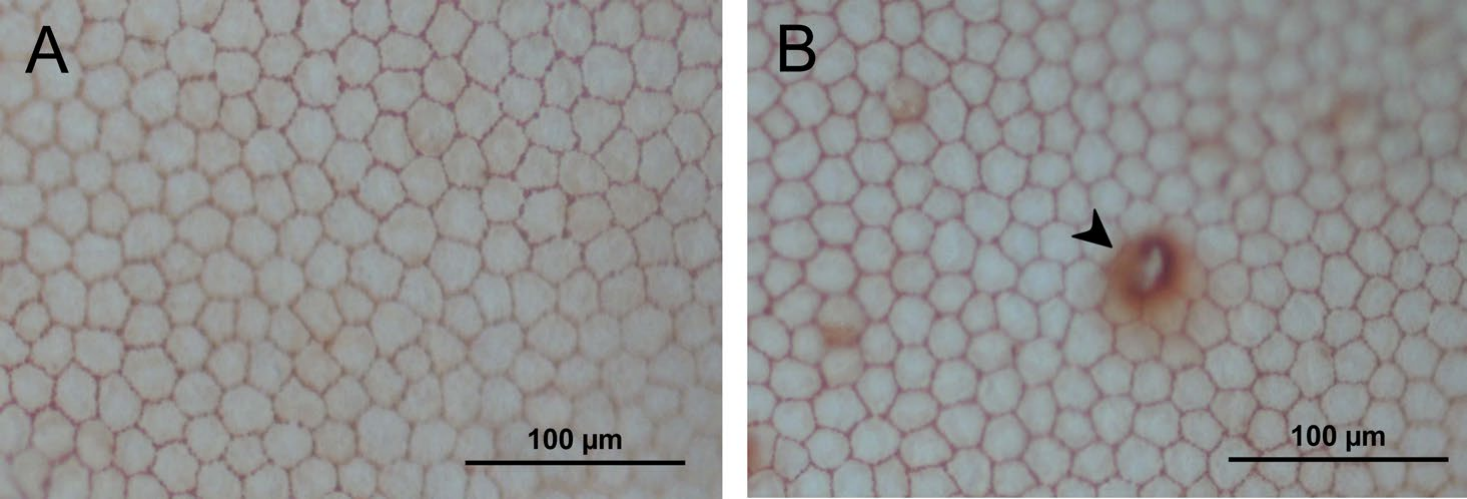
Representative images of the corneal endothelium from a 4‐year‐old cat, stained with trypan blue and alizarin red. Both panels (A, B) are from the same cornea at 20× magnification. Panel B shows a sporadic alizarin red precipitate (arrowhead), occasionally observed during staining.

The stained corneal endothelium was examined under light microscopy at 20× magnification immediately following tissue processing (Figure 2). Three representative images were captured per corneal sample. Image analysis was performed using ImageJ software (National Institutes of Health, version 1.54) to assess the following parameters:

- *Mean cell density* (MCD) in cell/mm^2^—Cells within a 20,000 μm^2^ rectangular region of interest (ROI) were manually counted, adhering to established border‐counting protocols [15]. MCD was calculated as the total cell count divided by the ROI size.
- *Mean cell area* (MCA) in μm^2^—Fifty cells were randomly selected per image, and their borders were traced manually. Cell area was measured in ImageJ, which automatically calculated the MCA and standard deviation.
- *Polymegathism* in %—Defined as the coefficient of variation of cell area, polymegathism was calculated by dividing the standard deviation of cell area by the MCA.
- *Pleomorphism* in %—Defined as the proportion of endothelial cells with a regular hexagonal shape, pleomorphism was determined by counting cells with six sides out of 50 randomly selected cells per image.

### 2.4 Data Analysis

Data normality was assessed using the Shapiro–Wilk test. Wilcoxon signed rank tests were used to compare results between the right and left eyes; since no statistical differences were observed for any outcome (*p* ≥ 0.117), the average of both eyes was used for further analysis. Outcomes were compared between male and female cats, as well as young (< 10 years old) and older (≥ 10 years old) cats, using Mann–Whitney rank sum tests. Associations between age and endothelial morphological features were evaluated using Spearman correlation tests, with interpretations based on standard guidelines [17]. No a priori sample size calculation was performed due to the lack of ex vivo effect‐size estimates at study onset. Post hoc justification based on the observed age‐morphology correlations indicated that the sample of 50 cats provided sufficient power to detect the associations for MCD (*r* = −0.653) and MCA (*r* = 0.593), and near‐conventional power for pleomorphism (*r* = 0.375) at *α* = 0.05 (two‐sided) and power of 80%. Statistical analyses were performed using SigmaPlot version 15.0 (Systat Software Inc.), with *p* values lower than 0.05 considered statistically significant.

## 3 Results

The study population consisted of 50 cats (*n* = 100 eyes) representing a variety of breeds, including Domestic Shorthair (*n* = 32), mixed breed (*n* = 10), British Shorthair (*n* = 4), Scottish Fold (*n* = 2), Domestic Longhair (*n* = 1), and Maine Coon (*n* = 1). Cats ranged in age from 0.1 to 15.5 years (mean ± SD, 8.1 ± 4.7 years; median, 10 years), and in body weight from 0.3 to 11.3 kg (mean ± SD, 3.9 ± 2.3 kg; median, 3.3 kg). All animals were enrolled post‐mortem (with owner consent) following death or euthanasia due to unrelated medical conditions, such as acute kidney injury or congestive heart failure (see Table S1 for details).

Data did not follow a normal distribution for any outcome (*p* ≤ 0.034). Table 1 summarizes the main morphometric findings. No statistically significant differences were observed between male and female cats for any outcome (*p* ≥ 0.061). In contrast, comparisons between age groups revealed that younger cats had significantly smaller endothelial cell area (*p* < 0.001), lower pleomorphism (*p* = 0.014), and higher endothelial cell density (*p* < 0.001) compared to older cats. Although polymegathism was lower in younger cats, the difference was not statistically significant (*p* = 0.587).

**TABLE 1:** Median ± SEM (minimum–maximum) of various corneal endothelium parameters in 50 cats post‐mortem.

	Endothelial cell area (μm^2^)	Endothelial cell density (cells/mm^2^)	Polymegathism (%)	Pleomorphism (%)
All	352 ± 10 (104–458)	2766 ± 196 (2250–10 183)	14 ± 0.3 (11–20)	85 ± 0.8 (60–94)
Male	341 ± 14 (104–455)	2875 ± 332 (2250–10 183)	14 ± 0.4 (11–18)	83 ± 1.3 (60–94)
Female	363 ± 13 (181–458)	2700 ± 139 (2333–5166)	14 ± 0.4 (12–20)	85 ± 0.9 (74–92)
Young (< 10 years old)	330 ± 16 (104–406)	2970 ± 385 (2366–10 183)	13 ± 0.4 (11–18)	82 ± 1.4 (60–92)
Older (≥ 10 years old)	367 ± 8* (300–458)	2625 ± 41* (2250–3133)	14 ± 0.4 (11–20)	86 ± 0.8* (79–94)

* Any significant differences (*p* < 0.05) between male versus female cats, and between young (< 10 years old) versus older cats (≥ 10 years old).

**FIGURE 3:**
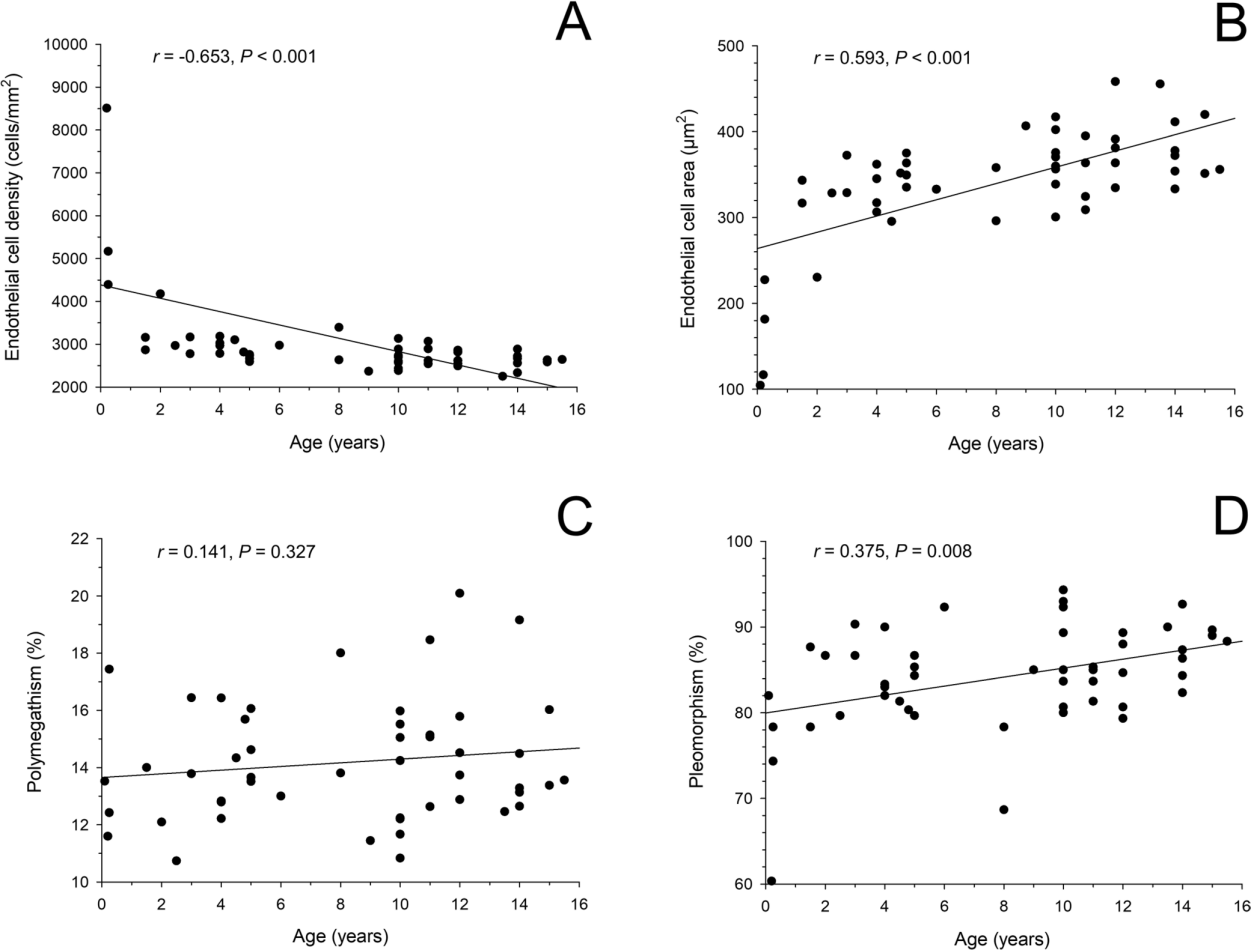
Spearman correlation between age and corneal endothelial parameters in 50 cats. Age was significantly associated with lower endothelial cell density (A) and larger endothelial cell area (B), while no correlation was found with polymegathism (C). A weak but significant positive correlation was observed between age and pleomorphism (D).

**FIGURE 4:**
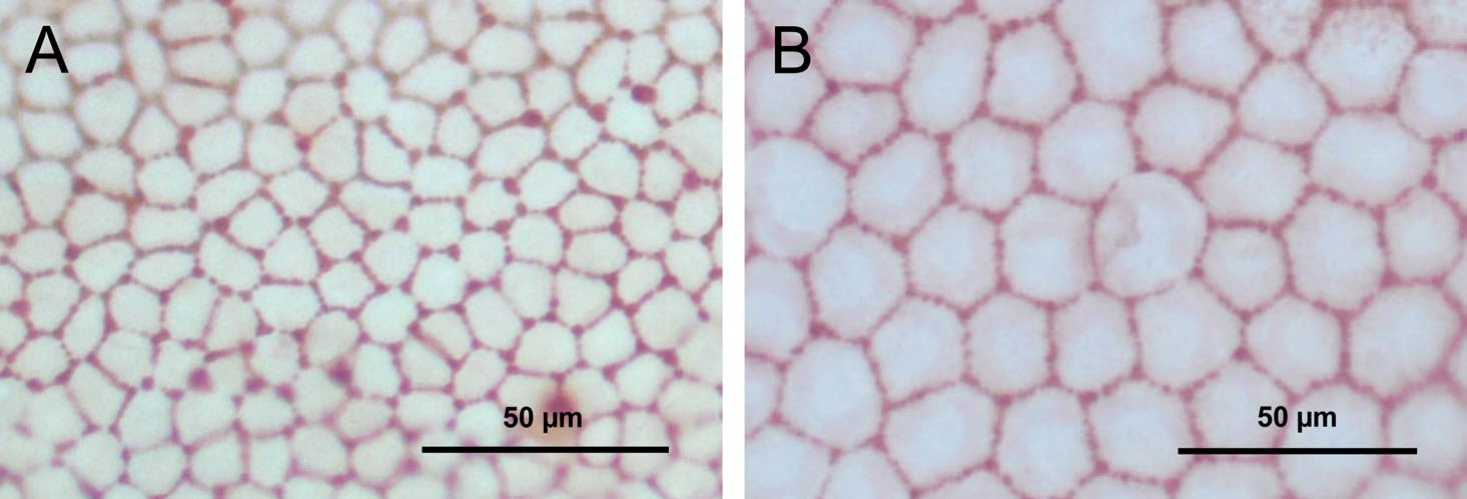
Representative images of stained feline corneal endothelium: (A) a 3‐month‐old kitten and (B) a 12‐year‐old cat. Both images were captured at 20× magnification and illustrate age‐associated morphological differences. Compared to the kitten (A), the older cat (B) shows reduced cell density, enlarged endothelial cells, and increased pleomorphism. These visual findings mirror the quantified age‐related changes described in the study.

Spearman correlation analysis further supported these findings (Figure 3), demonstrating a moderate positive correlation between age and endothelial cell area (*r* = 0.593, *p* < 0.001), a strong negative correlation between age and endothelial cell density (*r* = −0.653, *p* < 0.001), and a weak positive correlation between age and pleomorphism (*r* = 0.375, *p* = 0.008). No significant correlation was found between age and polymegathism (*p* = 0.327). Figure 4 provides representative images of the corneal endothelium from a young kitten and an adult cat, visually illustrating the observed age‐related differences.

## 4 Discussion

This study demonstrates that ex vivo analysis using vital dye staining and light microscopy enables clear, consistent evaluation of key feline corneal endothelial parameters, including endothelial cell density, cell size, polymegathism, and hexagonality. The technique also detects age‐related morphologic changes and provides a practical, cost‐effective tool for assessing endothelial health in settings where in vivo imaging may be impractical or unavailable.

A key methodological improvement in the current study was the adoption of an 8‐mm central corneal punch biopsy, which replaced the whole‐cornea flat‐mount technique used in a previous canine study [15]. This refinement improved tissue handling, reduced preparation time, and minimized tissue wrinkling, thereby enhancing overall imaging quality and throughput. When comparing species using the same staining and imaging protocols, feline corneal endothelial cells exhibited a higher average cell density (2766 cells/mm^2^) and smaller mean cell area (352 μm^2^) relative to dogs (2544 cells/mm^2^ and 431 μm^2^, respectively) [15]. Polymegathism was mildly lower in cats (14% vs. 17% in dogs), while pleomorphism was comparable (85% in cats vs. 84% in dogs). These findings suggest a denser and more compact endothelial mosaic in cats, which may reflect species‐specific differences in cellular architecture, metabolic demands, or age‐related remodeling dynamics.

Age‐related differences were evident in this ex vivo feline cohort, a pattern that mirrors human aging and underscores the translational relevance of the present findings. Older cats (≥ 10 years of age) had significantly larger endothelial cell areas (367 μm^2^ vs. 330 μm^2^) and lower MCDs (2625 vs. 2970 cells/mm^2^) compared to younger cats. Pleomorphism was slightly higher in the older group (86% vs. 82%), while polymegathism remained comparable between groups (13%–14%). These findings align with published in vivo data demonstrating age‐associated endothelial remodeling in cats and other species [10, 13], including increased cell size variability and loss of hexagonal morphology over time.

Feline corneal endothelial morphology has been previously characterized using several imaging modalities. Ex vivo specular microscopy of post‐mortem feline eyes, as performed by Franzen et al. (2010), revealed a clear age‐related decline in MCD from 6493 cells/mm^2^ in kittens to 2873 cells/mm^2^ in adult cats, with increasing cell area and pleomorphism [18]. In vivo specular microscopy, used in earlier studies by Chan‐Ling & Curmi (1988), Cohen et al. (1990), Bourne et al. (1994), and Bostan et al. (2016), reported adult MCDs generally ranging from 2395 – 3271 cells/mm^2^ [5, 11, 12, 14]. Kafarnik et al. (2007), using in vivo confocal microscopy, reported similar MCDs with 3038 cells/mm^2^ in juveniles and 2520 cells/mm^2^ in adults [13]. The present study employed ex vivo vital dye staining and light microscopy, yielding MCD of 2766 cells/mm^2^ (2970 in younger cats, 2625 in older cats), with polymegathism and pleomorphism values (14% and 85%) well within the ranges reported using other techniques. While direct comparisons between methodologies should be made cautiously, the morphometric values and trends identified in the present study align with these in vivo findings, supporting the validity of the ex vivo approach.

The feline corneal endothelium, like that of humans, has minimal mitotic capacity and compensates for cell loss primarily through enlargement and migration of remaining cells [6]. In both species, endothelial cell density can reportedly decline to approximately 800–1000 cells/mm^2^ before clinical decompensation and corneal edema develop [1, 19]. These shared features, along with other anatomical and physiological similarities, have positioned the cat as a valuable translational model for studying human endothelial dysfunction [4, 11, 14, 20, 21, 22, 23, 24, 25]. As such, the baseline values established in the present work could serve as an important reference for future studies evaluating disease progression, surgical impact, or therapeutic efficacy [3, 26]. The methodology may also facilitate objective evaluation of emerging therapies, including tissue‐engineered grafts, xenogeneic cell transplantation, and pharmacologic agents that promote endothelial preservation or repair [3, 20, 21, 26].

This study has some limitations. Although all eyes underwent slit‐lamp biomicroscopy prior to processing, cats were not examined pre‐mortem, intraocular pressure was not measured, and a few medical histories were incomplete. Therefore, mild or subclinical intraocular disease, glaucoma, or systemic factors influencing endothelial health cannot be entirely excluded; nevertheless, systematic slit‐lamp screening minimized the likelihood of overt anterior segment disease, supporting the overall reliability of the findings. Another limitation is that sample collection focused on the central 8‐mm region of the cornea, even though regional variability in endothelial density and function has been reported; future studies may therefore benefit from incorporating peripheral morphometry, particularly in diseased eyes where regional differences may be amplified. In addition, while this technique provides high‐quality morphometric data, it does not offer functional assessment of the endothelial barrier or pump. Future investigations should also integrate in vivo imaging modalities such as confocal or specular microscopy in the same subjects to enable direct methodological comparison with ex vivo staining and further strengthen interpretation of endothelial findings. Finally, complementary approaches such as immunohistochemistry, fluorophotometry, or electrophysiological assays in parallel models may enhance the overall utility of this platform [12, 14, 27].

In conclusion, the results of this ex vivo study were broadly consistent with previously reported in vivo data and demonstrate that this biopsy‐based method reliably captures key features of the feline corneal endothelium. Beyond its immediate relevance to feline ophthalmic research, the ex vivo platform described here provides a robust framework for comparative and translational research targeting corneal endothelial dysfunction. By leveraging the anatomical and physiological parallels between feline and human corneas, this methodology may accelerate the development and evaluation of interventions for age‐related or disease‐induced endothelial failure in humans.

## Ethics Statement

Animal owners or owners' representatives provided written consent for the use of the cadaver and/or tissues.

## Conflicts of Interest

The authors declare no conflicts of interest.

## Supporting information


**Table S1:** Demographics and causes of death or euthanasia in the study population (*n* = 50 cats).

## Data Availability

The data that support the findings of this study are available from the corresponding author upon reasonable request.
