# Supplementary material for: An Ex Vivo Morphometric Study of the Feline Corneal Endothelium (100 Eyes)
Source: Vet Ophthalmol. 2025 Oct 28;29(2):e70093. doi: 10.1111/vop.70093 (PMC12963510; doi:10.1111/vop.70093)
Supplement: Supplementary file 1 — Table S1: Demographics and causes of death or euthanasia in the study population (n = 50 cats). [file VOP-29-0-s001.docx]

**Supplementary Table 1.** Demographics and causes of death or euthanasia in the study population (*n* = 50 cats).

| **Cause of death or euthanasia** | **Weight  (kg)** | **Age (years)** | **Sex** | **Breed** |  |
| --- | --- | --- | --- | --- | --- |
| AKI, pancreatitis | 2.5 | 4.8 | FS | Scottish Fold | 1 |
| Head trauma, pneumothorax | 4.3 | 4 | MN | DSH | 2 |
| High-rise syndrome | – | 8 | MN | DSH | 3 |
| AKI, cardiogenic pulmonary edema | 3.3 | 11 | FS | DSH | 4 |
| Severe bite wounds | 7 | 1.5 | MN | Maine Coon | 5 |
| FIV | – | 12 | FS | DSH | 6 |
| End stage CKD | 4.3 | 10 | FS | DSH | 7 |
| Pleural effusion | 4 | 2.5 | MI | DSH | 8 |
| FIV | – | 4 | MI | DSH | 9 |
| FIP | 1.5 | 0.3 | FI | DSH | 10 |
| AKI, pancreatitis | 11.25 | 8 | MN | DSH | 11 |
| AKI | – | 4.5 | MN | DSH | 12 |
| Head trauma | 6 | 1.5 | MN | DSH | 13 |
| FIP | – | 5 | FI | DSH | 14 |
| High-rise syndrome | 5.8 | 12 | MN | DSH | 15 |
| Meningitis, suspected FIP | – | 0.3 | FI | DSH | 16 |
| AKI | 3 | 6 | FI | DLH | 17 |
| AKI | – | 14 | FS | DSH | 18 |
| Sepsis | 2.5 | 12 | FS | DSH | 19 |
| End stage CKD | – | 14 | FS | DSH | 20 |
| Ureteral obstruction | – | 10 | MN | British Shorthair | 21 |
| AKI, heart failure | 2.5 | 11 | MN | British Shorthair | 22 |
| Multiple congenital anomalies | 0.4 | 0.15 | MI | DSH | 23 |
| ATE | – | 12 | FS | DSH | 24 |
| High-rise syndrome | – | 5 | MN | DSH | 25 |
| High-rise syndrome, AKI | – | 4 | MI | DSH | 26 |
| End stage CKD | – | 14 | MN | DSH | 27 |
| FIP, peritonitis | – | 3 | FS | DSH | 28 |
| Brain tumor | – | 10 | MN | DSH | 29 |
| Hit by car | 2.3 | 2 | MI | DSH | 30 |
| AKI | 5.8 | 10 | MN | DSH | 31 |
| ATE | 6.4 | 3 | MN | DSH | 32 |
| Severe weakness | 0.3 | 0.1 | MI | DSH | 33 |
| AKI | – | 11 | FI | Scottish Fold | 34 |
| AKI | – | 15.5 | FI | DSH | 35 |
| Heart failure | – | 4 | MN | DSH | 36 |
| AKI, pyelonephritis | 2.6 | 10 | FS | DSH | 37 |
| Head trauma | – | 11 | FS | DSH | 38 |
| End stage CKD | 3.5 | 5 | MN | DSH | 39 |
| End stage CKD | 4 | 5 | FS | DSH | 40 |
| Anemia, thrombocytopenia | 3.3 | 10 | MN | DSH | 41 |
| AKI, pancreatitis | 2.8 | 15 | FS | British Shorthair | 42 |
| Severe anemia | 3.1 | 14 | FS | DSH | 43 |
| ATE | – | 10 | MN | DSH | 44 |
| Squamous cell carcinoma | – | 14 | FS | DSH | 45 |
| ATE | 7.7 | 9 | MN | DSH | 46 |
| End stage CKD | – | 10 | MN | DSH | 47 |
| AKI, ATE | 4.6 | 13.5 | MN | DSH | 48 |
| AKI, heart failure | 2.7 | 15 | FS | British Shorthair | 49 |
| Oral cavity fibrosarcoma | 2.5 | 12 | FS | DSH | 50 |

MI = Male intact; MN = Male neutered; FI = Female intact; FS = Female spayed; DSH = Domestic Shorthair; DLH = Domestic Longhair; AKI = Acute kidney injury; CKD = Chronic kidney disease; FIV = Feline immunodeficiency virus; FIP = Feline infectious peritonitis; ATE = Aortic thromboembolism. – Not reported.
